# Efficacy of Carfilzomib, Lenalidomide, and Dexamethasone for Extramedullary Intracranial Localization of Multiple Myeloma

**DOI:** 10.1155/2018/2312430

**Published:** 2018-11-29

**Authors:** Giuseppe Mele, Domenico Pastore

**Affiliations:** Department of Haematology and BMT Unit, Antonio Perrino Hospital, Brindisi, Italy

## Abstract

EMD of myeloma usually occurs several years after diagnosis and is associated with a very poor OS of <6 months due to the fact that there are no efficient treatment options. In rrMM with EMDs, the most effective treatment is a lymphoma-like polychemotherapy regimen such as PACE, Dexa-BEAM, and HyperCVAD followed by ASCT or allogeneic SCT. RT of soft-tissue plasmacytoma is the further treatment choice and results in a high rate of local control and a prolonged disease-free survival. We report the case of a 41-year-old man affected by ultra-high-risk symptomatic IgA*λ* MM with extramedullary intracranial soft-tissue relapsed after VTD-PACE followed by ASCT. The salvage program with KRd regimen determines a second biochemical and haematological remission and a gradual reduction in size of the extramedullary intracranial soft-tissue even in the absence of local aggressive radiotherapy, suggesting that carfilzomib and lenalidomide together could be effective also in this critical situation.

## 1. Introduction

Extramedullary dissemination (EMD) of myeloma usually occurs several years after initial diagnosis or can be present at the time of initial diagnosis of myeloma. Any organ or system can be affected. At the time of diagnosis, EMD is associated with an adverse prognosis, while at the time of relapse, EMD is associated with a very poor overall survival of <6 months [1] due to the fact that there are no efficient treatment options for these patients.

## 2. Materials and Methods

Here follows is the case of a 41-year-old man with “de novo” ultra-high-risk symptomatic IgA*λ* multiple myeloma (MM) diagnosed in March 2017. Biochemistry tests showed elevated levels of monoclonal spike (7.2 g/dL), serum *λ* free light-chain (750 mg/L), and LDH. A bone marrow examination showed a massive infiltration (90%) of immature plasma cells (PCs). FISH analysis revealed high-risk cytogenetic abnormalities (gain (1q); *t*(4;14)). MR imaging (MRI) showed soft tissue in the right intraorbital region. A PET-CT scan revealed an intensive diffuse fluorodeoxiglucose avidity and several bone lesions; no further extramedullary lesions were detected by PET-TC. The prognostic analysis showed a stage III R-ISS (revised International Staging System). A combined intensive lymphoma-like regimen as VTD-PACE (bortezomib 1 mg/m^2^ as subcutaneous injection on days 1, 4, 8, and 11; thalidomide 50 mg daily; dexamethasone 40 mg/d p.o., days 4–7; cisplatin 10 mg/m^2^/d, days 4–7; doxorubicin 10 mg/m^2^/d, days 4–7; cyclophosphamide 400 mg/m^2^/d, days 4–7; etoposide 40 mg/m^2^/d, days 4–7) and zoledronic acid were given. A complete biochemical response was obtained following two cycles; serum protein electrophoresis showed no evidence of the M-component. Autologous peripheral blood stem cell transplant (ASCT), conditioned with melphalan 200 mg/m^2^, was performed in August 2017.

**Figure 1:**
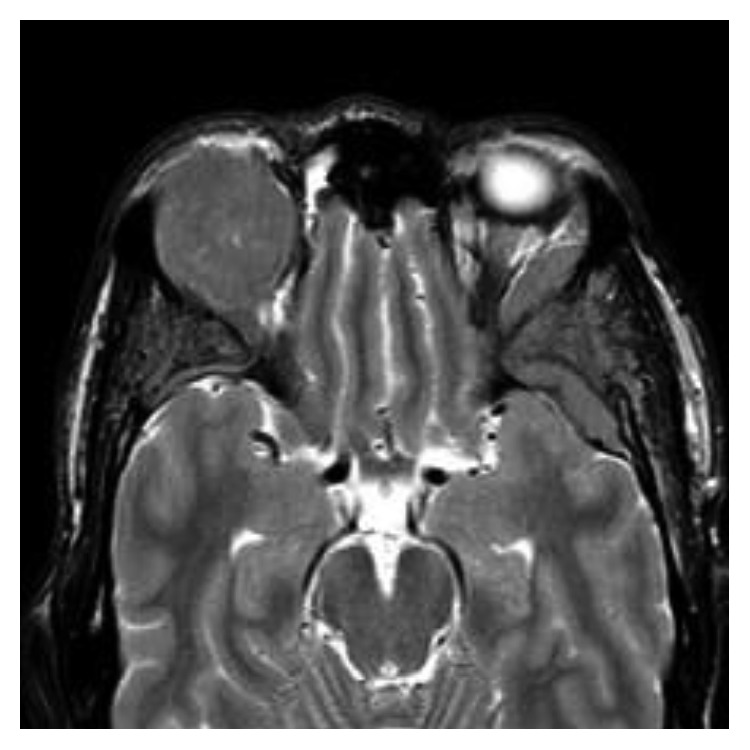
MRI before KRd treatment: increase in size of soft tissue in the right intraorbital region, pressing against the upper and lateral rectus muscle; the same soft tissue was observed in the extrassial site close to the large sphenoid wing, pressing against the left temporal muscle.

**Figure 2:**
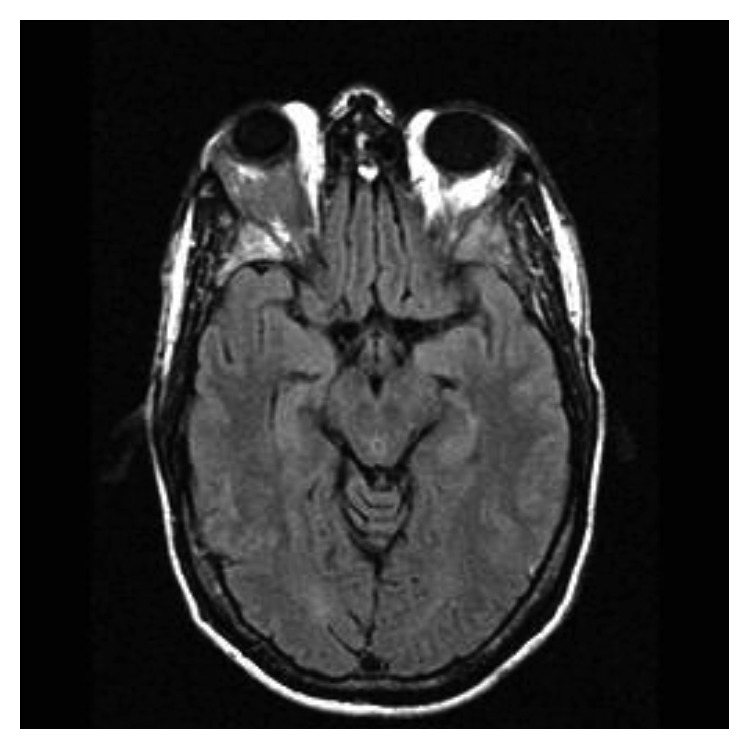
MRI after KRd treatment: gradual reduction in size of the soft-tissue plasmacytomas in the absence of local RT.

One month later, in September 2017, the patient was admitted to our hospital after suffering for four days with blurred vision in one eye and right exophthalmos. An MRI showed increase in size of soft tissue in the right intraorbital region, pressing against the upper and lateral rectus muscle; the same soft tissue was observed in the extrassial site close to the large sphenoid wing, pressing against the left temporal muscle (Figure 1). The MRI did not show leptomeningeal enhancement or CNS involvement. The M-component was present in serum and urine. The percentage of bone marrow PCs was 80%. The cerebrospinal fluid analysis using both cytology and flow-cytometry did not show plasmacytosis. As a symptomatic relapse, the patient received a salvage program with KRd regimen (carfilzomib on days 1-2, 8-9, and 15-16 (starting dose 20 mg/m^2^ on days 1 and 2 of cycle 1, target dose 27 mg/m^2^ thereafter); lenalidomide 25 mg on days 1–21; oral dexamethasone 40 mg before each dose of carfilzomib, on days 1, 8, 15, and 22 of a 28-day cycle). The treatment planning of radiotherapy (RT) required an optimal immobilization with 3-point thermoplastic mask necessary to allow tight margins to spare the organ at risk (chiasm, contralateral orbit, and left optic nerve), but the patient continued to refuse RT due to possible severe neurological complications. After 4 cycles, the clinical evolution was favorable with the disappearance of symptoms, achieving a second biochemical and haematological complete remission. In fact, in December 2017, no clonal PCs with plasmablastic morphology were detected in bone marrow or peripheral blood; serum and urine protein electrophoresis showed no evidence of the M-component; serum free light-chain ratio was normal. A new MRI showed a gradual reduction in size of the soft-tissue plasmacytomas, in the absence of local aggressive RT (Figure 2).

Treatment tolerance was excellent. The patient had adequate hepatic and renal function. During cycles 1 and 2 (C1 and C2), the most common G3-G4 adverse events were thrombocytopenia (PLT count <30 × 10^3^ *µ*l) and neutropenia (absolute neutrophil count <0.5 × 10^3^ *µ*l). There were no bleeding events associated with thrombocytopenia and no pneumonia/upper respiratory infections associated with neutropenia. From C2D1, KRd was administered at the maximum planned dose (carfilzomib 20 mg/m^2^ on days 1-2, 8-9, and 15-16; lenalidomide 15 mg on days 1–21; oral dexamethasone 40 mg on days 1, 8, 15, and 22 of a 28-day cycle) due to thrombocytopenia and neutropenia. From C3D1, carfilzomib dose was escalated to 27 mg/m^2^ for all subsequent cycles. At present, the patient is still alive and continues to receive the 8th cycle of treatment, preserving the second remission. Because of the inability to cure these forms of ultra-high-risk MM with currently available therapies and because of lack of durable disease control, and in the absence of a sibling donor, we started the search for an HLA-identical unrelated adult donor.

## 3. Discussion

EMD is uncommon in frontline MM, but it is common in advanced disease stages. EMDs are extraordinarily heterogeneous, and their management is particularly difficult due to the fact that all current treatment approaches are unsatisfactory. For “de novo EMDs” in patients eligible for SCT, prospective studies suggest that a triplet induction therapy bortezomib-based such as VTD or PAD followed by tandem ASCT, a triplet consolidation therapy, and a maintenance treatment [2, 3] constitute the best upfront approach. In relapsed/refractory patients with EMDs, the most effective treatment is a lymphoma-like polychemotherapy regimen such as PACE, Dexa-BEAM, and HyperCVAD [4, 5] followed by ASCT or allogeneic SCT whenever possible. RT of soft-tissue plasmacytoma (>40 grays) is the further treatment of choice and results in a high rate of local control and a prolonged disease-free survival [6].

At the time of diagnosis, our patient, having an ultra-high-risk disease, had been treated with a combined intensive regimen (VTD-PACE) followed by ASCT. At the time of early relapse with EMD, obviously, our hope was the identification of novel agents with new mechanisms and new strategies for the treatment of this very aggressive form of MM. Novel approaches are warranted to improve response and overall survival. Currently, there is no rationale to favor a new specific therapeutic class (IMIDs, PI, or monoclonal antibody) over another, and the data concerning the efficacy of newer drugs are very limited. In fact, there are not sufficient clinical data on the efficiency of new IMIDs such as lenalidomide and pomalidomide, on the benefit of new proteasome inhibitors such as carfilzomib or ixazomib, or on the curative potential of newer agents such as monoclonal antibodies.

The positive results obtained in patients with advanced and refractory disease and the good safety profile and tolerability make carfilzomib one of the most important recent options in the treatment of relapsed-refractory MM [7]. Of interest, preclinical studies [8] show that PR-171 is rapidly cleared from the plasma compartment following i.v. administration, suggesting extensive penetration into peripheral tissue. Its astonishing ability for rapid spread and penetration into peripheral tissue and its potential therapeutic impact on EMDs convinced us to choose and use a carfilzomib-based triplet combination. In our case report, the patient obtained a reduction in size of the extramedullary intracranial soft-tissue even in the absence of local aggressive radiotherapy, thus suggesting that carfilzomib and lenalidomide together could be effective also in this rare and critical situation. More clinical studies and more long-term follow-up are required to establish the role of combined regimen KRd in the control of EMDs.
